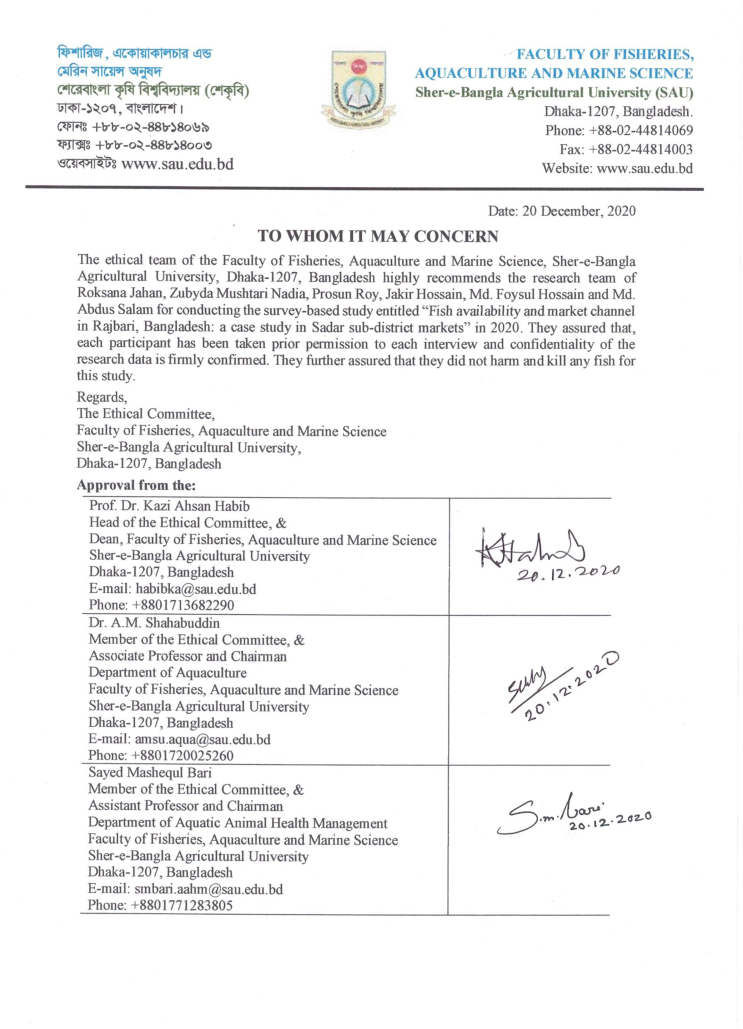# Fish availability and market channel in Rajbari, Bangladesh

**DOI:** 10.1016/j.heliyon.2022.e10526

**Published:** 2022-09-06

**Authors:** Zubyda Mushtari Nadia, Prosun Roy, Jakir Hossain, Md. Foysul Hossain, Mofasser Rahman, Md. Abdus Salam, Roksana Jahan

**Affiliations:** a Department of Aquatic Animal Health Management, Sher-e-Bangla Agricultural University, Dhaka 1207, Bangladesh; b Department of Aquaculture, Bangladesh Agricultural University, Mymensingh 2202, Bangladesh; c Department of Marine Fisheries and Oceanography, Sher-e-Bangla Agricultural University, Dhaka 1207, Bangladesh; d Department of Aquatic Environment and Resource Management, Sher-e-Bangla Agricultural University, Dhaka 1207, Bangladesh; e Department of Agribusiness and Marketing, Sher-e-Bangla Agricultural University, Dhaka 1207, Bangladesh

**Keywords:** Fisheries traders, Processed fish, Margin, Value chain, AHP analysis

## Abstract

Knowledge about fisheries market margins and fish availability in the market is crucial to establish an effective and well-planned marketing strategy. Hereby, the study was conducted to bring some knowledge on the availability of raw and processed fish and its marketing channel in Rajbari Sadar, Bangladesh from June to December 2020. Here, data were collected from fish traders and consumers of the target markets through questionnaire surveys, focus group discussions and field visits. The study found 107 fish and shellfish species in these markets, of which more than 50% species were commonly available and less than 10% were found very rarely. The study also observed 18 types of processed fish products in these markets including dried (77.77%), salted (16.67%) and fermented (5.56%) products arriving from mostly Dhaka and Chattogram. The markets were dominated by wild freshwater fishes of nearby rivers, ponds and canals etc. The length of processed and marine fish marketing channel was comparatively longer than freshwater one because these items are supplied here from coastal districts via several intermediaries. The study revealed remarkable market margin for hilsa fish even noticed up to 57.14% at consumer level. There was also observed some major constraints to a good marketing system such as unplanned market location, insufficient drainage system, high transportation cost, etc. Based on the constraints, the study would suggest to establish a well-planned and modern equipped fish market with high quality cold storage and ice factories, which could help to ensure smoother transaction route from production to customer minimizing economic loss.

## 1 Introduction

As a substantially up-growing sector of Bangladesh, fisheries have an enormous prospect to become a sustainable economic wing since it is very rising sector contributing 3.50% of the total GDP and ranked 4^th^ in global aquaculture production (DoF, 2020). The viability of this sector is interlinked with some fisheries related factors such as fisheries biodiversity, fish availability, fish production and its marketing systems, fisheries personnel, institutional infrastructures and developmental facilities etc. Among these, fish market and marketing system imply two-way approach where both producers and consumers are involved. Organized market system and market structure are required to make fish available to consumers at the appropriate time and in the right place (Aura et al., 2019; Kamaylo et al., 2021). Fish marketing system is predominantly dependent on private ownership activities and operated through an interconnected process of some sequential events like fish farmers, fishermen or fish landing centers, local or village markets, township markets, gathering points, wholesale and retail markets (DoF, 2012). This sequential system of the market is known as marketing channel which ranges from production sector to consumer sector with some intra- and inter-linkage intermediaries or middlemen.

In Bangladesh, fish marketing system includes the livelihoods of a large number of people (more than 17 million) linked with fish production, processing and packaging and the supply channel (BFTI, 2016). The typical fish market displays a common scenario of disorganized activities, thereby controlling some influential persons of the area, additionally involving a wide range of social, economic and political factors (Rashid, 2006). The fishermen and fish farmers are often bound to sell their products to the traders (Aratdar or Paikar) at a so-called price fixed by these middlemen. The fishermen and fish farmers cannot bargain against the ill price of their products set by the immediate middlemen because of their dependency on them for financial flows and illiteracy to raise voice for the proper pricing of their products (Aswathy et al., 2014; Kamaylo et al., 2021). Consequently, the fishing communities are particularly getting dominated by the immediate intermediaries as the middlemen have developed a regulated marketing chain absorbing the marginal fishing communities by determining a restricted pricing policy through the intermediaries at different levels of the marking chain. The marginal remote communities are going through the most serious marketing difficulties owing to lack of economic solvency, poor transportation facilities, insufficient storage facilities and adequate fisheries knowledge (Rahman, 1997). Most of the cases, the middlemen or intermediaries are dominating for achieving highest profits from the marketing channel (Bryceson, 1993). Sustainable aquaculture production depends on the producers getting sufficient profit, health and quality product, consumers demand and international competitiveness (Muir et al., 1995). On the other hand, consumers are looking for high-quality items at reasonable pricing. So, it is very important for the marketer to pick which route or channel is ideal for fish and products flow maintaining the quality and affordable price. Thus, determining the appropriate prices and market margins in the fish marketing channel will help to establish sustainable market policies ensuring a balanced profit margin for producers and all the intermediaries linked with the marketing system (Kaygisiz and Eken, 2018; Kamaylo et al., 2021; Sambuo et al., 2021).

Study on fish market aids in determining sufficient profits, suitable channel, market price and in lowering the degree of risk in making plans which are crucial for the aquaculture sustainability (Aura et al., 2019; Das et al., 2020; Rahman and Islam, 2020). Some studies have been investigated to explore the fish marketing system, marketing channel and the socio-economic environment of the traders in different areas of Bangladesh namely Gazipur (Debnath et al., 2019), Rajshahi city (Asaduzzaman et al., 2010), Paikgachha (Roy et al., 2020a) and Chittagong (Khalil et al., 2017). These studies explain the fisheries marketing systems in these regions and tried to find out suitable marketing strategies for those region. Marketing practice, fish demand, products types also vary on the basis of geographical location of the region and surrounding fisheries resources. For instance, in Bangladesh, Mymensingh region is well known for aquaculture where the price of culture fish is lower than the other region and the fishes are sent to other distant regions (FRSS, 2017; Uddin et al., 2019). Thus, marketing channel must be varied region to region. Rajbari is also a potential fisheries area because it is an agro-based riverine district, enriched in wild diversified fish species, located at the bank of Padma River, and has great potentiality for urbanization. Moreover, the region is also blessed with other small rivers, seasonal waters like beels those are great sources of fish (BBS, 2011; Nadia et al., 2021). But there is no study conducted yet in Rajbari District regarding fisheries marketing system. So, to flourish fisheries sector in this region, knowledge about current fisheries marketing systems, constraints and prospective potentialities is necessarily needed which is lacking in this region. Therefore, the study aimed to explore the fish marketing channel, value chain, marketing margin, fish availability in the markets and problems associated with fish marketing system in Rajbari Sadar, Bangladesh. For the first time, the study would provide an idea about the availability of fish, shellfish, marine and processed fish in the markets along with its marketing channel, and margins for each item. Based on this knowledge further intensive study could be conducted to find out sustainable profitable marketing channel and margins of both local and long distant fishery products in this region.

## 2 Materials and methods

### 2.1 Study area

**Figure 1:**
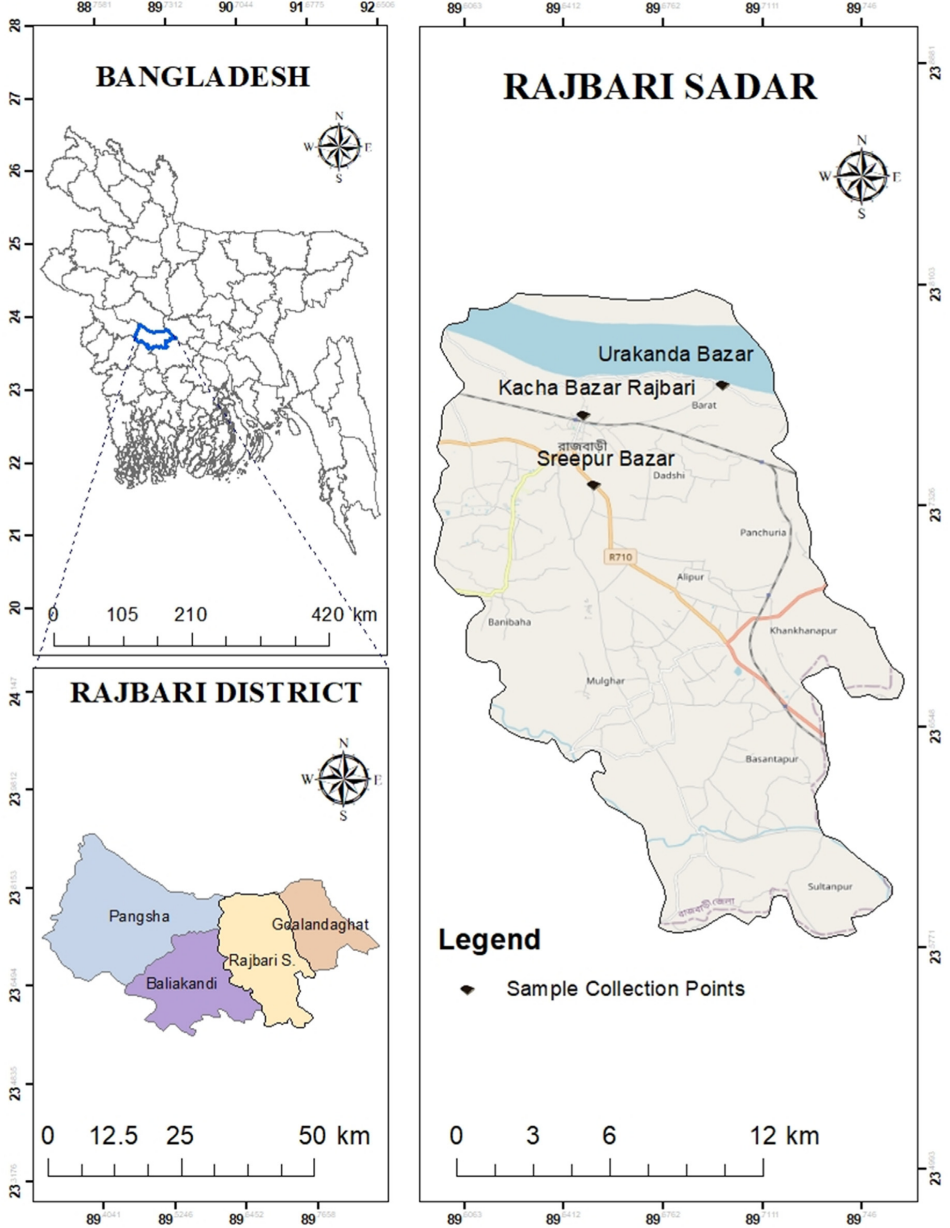
Map showing the study area, the district of Rajbari in Bangladesh (Source: ArcGIS software, version- 10.7.1).

Rajbari Sadar is one of the sub-districts of Rajbari and more than 75,000 households reside in this area (BBS, 2011). Three river-flows and more than six thousand aqua-ponds were documented in this sub-district earlier by BBS (2011). This study was conducted in three main fish retail markets of Rajbari Sadar namely, Kacha Bazar (with the coordinates across 23°45′54.9″N and 89°38′52.8″E), Sreepur Bazar (with the coordinates across 23°44′25.8″N and 89°39′06.1″E) and Urakanda Bazar (with the coordinates across 23°46′32.2″N and 89°41′49.3″E) (Figure 1) from June to December, 2020. The location of the research area was mapped using ArcGIS software (version 10.7.1). During the study, total fish traders were found 42, 33 and 28 in Kacha Bazar, Sreepur Bazar and Urakanda Bazar, respectively. Freshwater fish, marine fishes, and processed fish products are available in these markets.

### 2.2 Data collection method

Fundamental data of the market including available species, availability, demand, type, retail status, value additional activities, price and existing problems were collected using questionnaire interview including focus group discussion (FGD), field visit, and crosscheck interviews with fifteen key informants (KI) such as Upazila Fisheries Officer (UFO) and NGOs (ACI, BRAC, and Winrock International) staffs (Table 1). Sixty fish traders (20 from each market) were randomly chosen for questionnaire interview from the selected markets (Roy et al., 2020a). Besides, retail and wholesale prices, the traders also provided average price of fish and products at different stages of market channel through their experiences, data-book and contacting other fellow traders. Six focus group discussion (FGD) sessions of the participants were performed having 10–13 members in each group maintaining social distance and other health protocols of World Health Organization regarding COVID-19 as the study was conducted during COVID-19 pandemic. In addition, 20 consumers were interviewed at the market, home and their working places throughout the study period. The themes were identified and classified into manageable categories of different variables.

**Table 1:** An overview of empirical data collection methods.

Tools	Participants	Sample size	Research issues
Individual interview	Fish retailers	60	Market channel, value chain, species type (availability, demand, retailing status and price)
	Consumers	20	Species type (availability, demand, retailing status and price)
Focus group discussion (FGD)	Fish retailers, consumers	5 sessions of 10–13 members	Basic information and data on fish market species and market problems
Key informant interview (KII)	Government officer (UFO) and NGOs staff (ACI, BRAC, and Winrock International)	Government officer = 5 NGO staff = 10	Cross-checking of the collected data

Source: Author survey, 2020

#### 2.2.1 Species identification

Identification of the raw fish and shellfish species with their respective orders was carried out following Rahman (2005) and Roskov et al. (2017). The species, those were found difficult to identify during survey, were preserved in 10% buffered formalin and transported to the laboratory of the Department of Fisheries Biology and Genetics, Sher-e-Bangla Agricultural University, Bangladesh and samples were confirmed based on the morphometric and meristic appearances according to Talwar and Jhingran (1991) and Rahman (2005). Moreover, group of each species was specified from Froese and Pauly (2020).

#### 2.2.2 Availability, demand, origin, water-body and retail status

Several qualitative data were collected from the retailers on fish and shellfish stock in the markets like fish availability, consumers demand for each species, and selling status by the retailers.

Freshwater species indicates the species harvested from freshwaters like river, ponds, floodplains and beels. Marine species means the saltwater species and exotic species are the non-native species (Shamsuzzaman et al., 2017).

For representing the availability of fishes in the market, four categories were used such as “very rare”, “rare”, “few” and “common” according to Roy et al. (2020a). The term common indicates the species available throughout the year in the market. Whereas, availability of the stock in approximately 50, 30 and 10% period of the year was indicated by few, rare and very rare terms, respectively.

For demonstrating the demand of fish and processed products, three categories were used referred as- “low (<5%)”, “medium (10–20%)” and “high (>50%)”. In case of studying the origin of species, “capture”, “culture” and “both” categories were used. Capture fishes are harvested from natural waters like rivers, floodplain and sea; on the other hand culture fishes are from fish ponds (Shamsuzzaman et al., 2017; Roy et al., 2020b).

The retailing status of species was indicated using head namely “single” and “mixed” (Roy et al., 2020a). Moreover, several species are sold as both individually and mixture depending on the customer requirement and for the stocks “both” term is used as a retailing status.

#### 2.2.3 Market channel and value chain

In the fish marketing channel, different terminologies were used. “Fishers or farmers” work at the initial stage of market channel where fishermen catch fish from the natural water-body and fish farmers harvest fish from their culture ponds. “Auctioneer” means a person who sells fish on behalf of fish farmers and collectors and gets commission fees. “Wholesaler” usually procures fish from the assembly markets or buys directly from fish farmer and sells directly to retailers. “Retailer” is the person who buys fish and shellfish from wholesale fish dealers, undertakes limited processing activity and sells only to the ultimate consumer (Porras et al., 2017).

Porter (1985) introduced the notion of value chain, which covers the whole range of activities necessary to convey fish from the various stages of production to the end customers. In the chains different actors perform different value additional activities and enablers support, manage and monitor the functions. In the present study, value chain was mapped based on present findings following Porter (1985) and Uba et al. (2020).

#### 2.2.4 Price and market margin

Retail price of the fresh and processed products has been categorized into 4 groups such as “low”, “medium”, “high” and “very high” price indicates $1–3, $4–8, $9–12 and above $12, respectively. Roy et al. (2020a) in their study, grouped the fish from market into four distinctive category on the basis of their price.

“Raw” or “fresh fish” indicates both freshwater and marine species in the study. Market margin is the percentage of the final weighted average selling price taken by each stage of the marketing chain. The margin covers the costs involved in transferring from one stage to the next providing a reasonable return to those doing the marketing activities (Crawford, 1997). The market margin of fishers and different intermediaries is calculated using the following formula (Shepherd, 2007) shown as Eq. (1):

(1) $$\text{Market ​margin ​}\left(\text{\%}\right)\text{ ​}=\text{ ​}\frac{\text{Selling ​price ​}-\text{ ​Purchase ​price}}{\text{Selling ​price}}\times 100$$

#### 2.2.5 Constraints comparison and ranking

The weight for each constraints was determined by pair-wise comparisons in the context of a decision-making process known as the Analytic Hierarchy Process (AHP). It is used to compare the problems faced by the participants in the studied fish markets following Saaty (2005); Huo et al. (2011); Lokare and Jadhav (2016); and Chaibate et al. (2021).

### 2.3 Data evaluation and analysis

**Figure 2:**
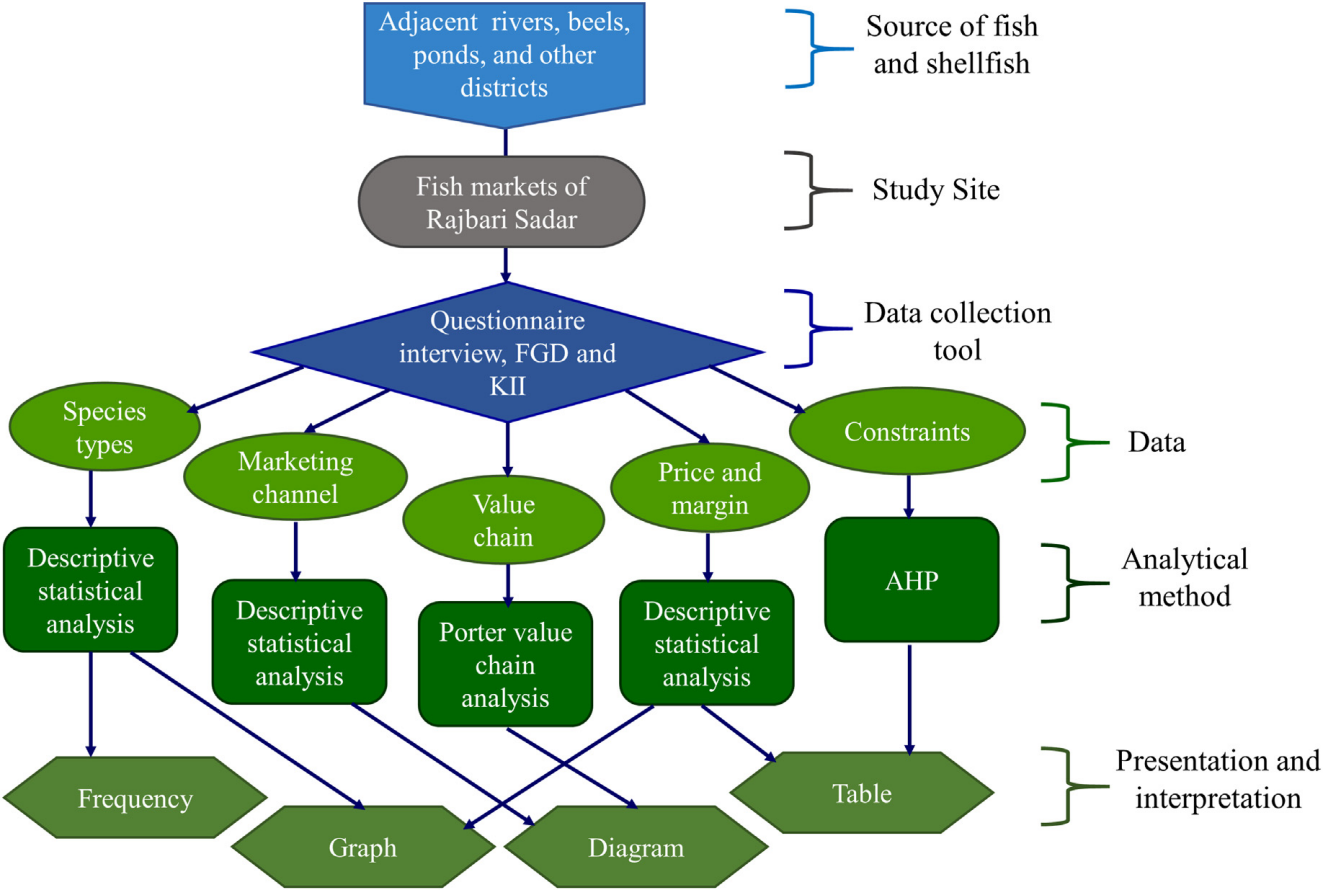
A conceptual framework on research methodology (Here, FGD: Focus Group Discussion, KII: Key Informant Interview and AHP: Analytic Hierarchy Process).

Through the interviews, a range of qualitative information and quantitative data was obtained from the participants. To acquire a conceptual grasp of this study, the data summary was arranged and analyzed. The quantitative data was formulated in MS Excel (version 2016) using descriptive statistics in frequencies and percentages. Graphs, tables, and flowcharts were used to represent the information (Figure 2).

#### 2.3.1 Descriptive data analysis

Categories of fish and shellfishes on the basis of scientific orders, group, type, availability, demand, retail status and price were calculated as frequency and percentage. Basic method of frequency percentage was employed following Roy et al. (2020a) and Sunny et al. (2021).

#### 2.3.2 AHP analysis

In order to set up the vector for the constraints, *n* × *n* pairwise comparison matrix *A* was formed following Saaty (2005) and Chaibate et al. (2021) which is given as Eq. (2):

(2) $$\text{A ​}=\text{ ​}\left(\begin{array}{lll} a_{11} & \cdots & a_{1n}\\ \vdots & \ddots & \vdots \\ a_{n1} & \cdots & a_{nn} \end{array}\right)={\left(a_{ij}\right)}_{ij^{’}}$$

Here, *a*_*ij*_ is the component of row *i* column *j* of the matrix, *n* is the number the evaluated constraints, *a*_*ji*_ = (1/*a*_*ij*_), and *a*_*ii*_ = *a*_*jj*_ = 1 and *a*_*ij*_ indicates the seriousness of the constraints *i* when compared to *j*.

Here, the relative seriousness between two problems is evaluated on the basis of a numerical scale ranging from 1 to 9. According to Saaty (2005), 1 indicates equal importance; 3 indicates moderately preferred; 5 indicates strongly preferred; 7 indicates very strongly preferred; 9 indicates extremely preferred; 2, 4, 5, 8 indicate intermediate values and reciprocals are used for inverse comparisons. After arranging the matrix *A*, the priority vector of constraints is calculated using normalized pairwise comparison matrix *A*_*norm*_ (written as Eq. 3) which is the total of the entries of each column is equal to 1 (Saaty, 2008; Huo et al., 2011; Chaibate et al., 2021).

(3) $${\text{A}}_{\text{norm}}\text{ ​}=\text{ ​}\left(\begin{array}{lll} \bar{a_{11}} & \cdots & \bar{a_{1n}}\\ \vdots & \ddots & \vdots \\ \bar{a_{n1}} & \cdots & \bar{a_{nn}} \end{array}\right)={\left(\bar{a_{ij}}\right)}_{ij^{•}}$$

The matrix *A*_*norm*_ entries $\bar{a_{ij}}$ are measures using the entries *a*_*ij*_ of the matrix *A* as the following Eq. (4) (Chaibate et al., 2021):

(4) $$\bar{a_{ij}}=\frac{a_{ij}}{\sum_{k=1}^{n}a_{kj}}$$

After that, the priority vector or Eigen vector (*P*) of constraints are calculated. Ranking of the constraints are made based on the values of Eigen vector; higher value of Eigen vector indicates more serious problem than other problems. It is calculated by averaging the values of each row of the matrix *A*_*norm*_ (Lokare and Jadhav, 2016; Chaibate et al., 2021) as Eq. (5):

(5) $$p_{i\;}=\frac{\sum_{k=1}^{n}\bar{a_{ik}}}{n}$$

In AHP, consistency ratio (CR) is calculated using Eq. (6) to evaluate the consistency of the priority made by the participants as follows (Lokare and Jadhav, 2016):

(6) $$\text{CR ​}=\text{ ​}\frac{\text{CI}}{\text{RI}}$$

When, 0 ≤ CR ≤ 0.1, the evaluations made by the participants are consistent. If CR > 0.1, the judgment made by the participants is inconsistent; CI is the Consistency Index evaluated using following formula (Eq. 7):

(7) $$\text{CI ​}=\text{ ​}\frac{\left(\text{λ}-\text{n}\right)}{n-1}$$

Here, λ is evaluated as the following Eq. (8):

(8) $$\lambda \text{ ​}=\text{ ​}\overset{n}{\underset{i=1}{\sum }}p_{i\;}*\text{ ​}\overset{n}{\underset{k=1}{\sum }}a_{ki}$$

RI is random index changes on the basis of *n*. Value of RI are taken from Chaibate et al. (2021).

## 2.4 Ethical statement

Prior to the survey, participants' permission was considered. Before initiating the survey, all of the respondents were informed about the principal goal and possible benefits of the research. All participants’ willingness for this study and anonymity have been assured as well as confidentiality of each interview was strictly maintained. The formal ethical agreement for this study was received from the ethical committee of Faculty of Fisheries, Aquaculture and Marine Science; Sher-e-Bangla Agricultural University, Dhaka, Bangladesh.

## 3 Results

### 3.1 Available fish and shellfish species

**Figure 3:**
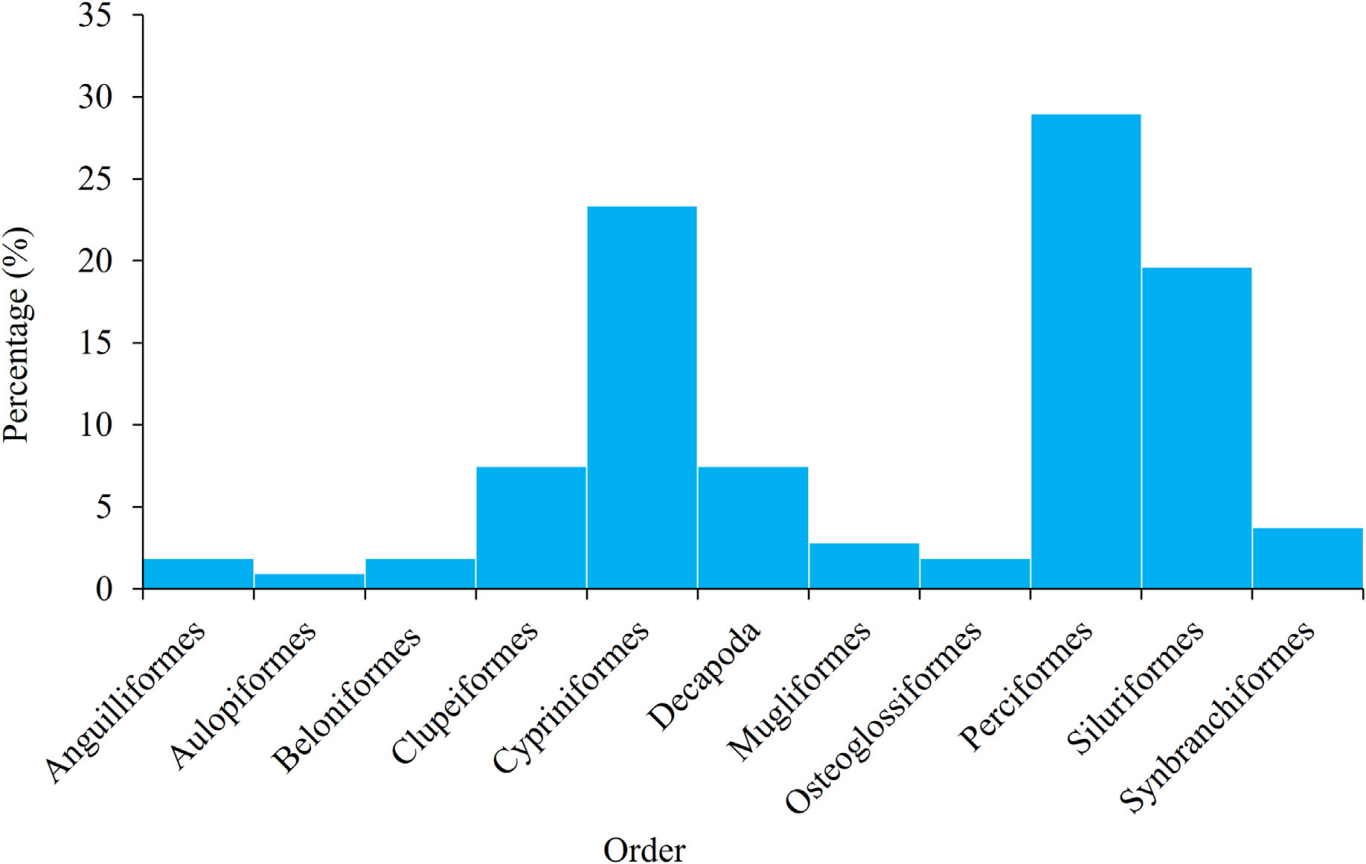
Orders of fish and shellfishes in the Rajbari fish markets (Source: Authors Survey, 2020).

**Figure 4:**
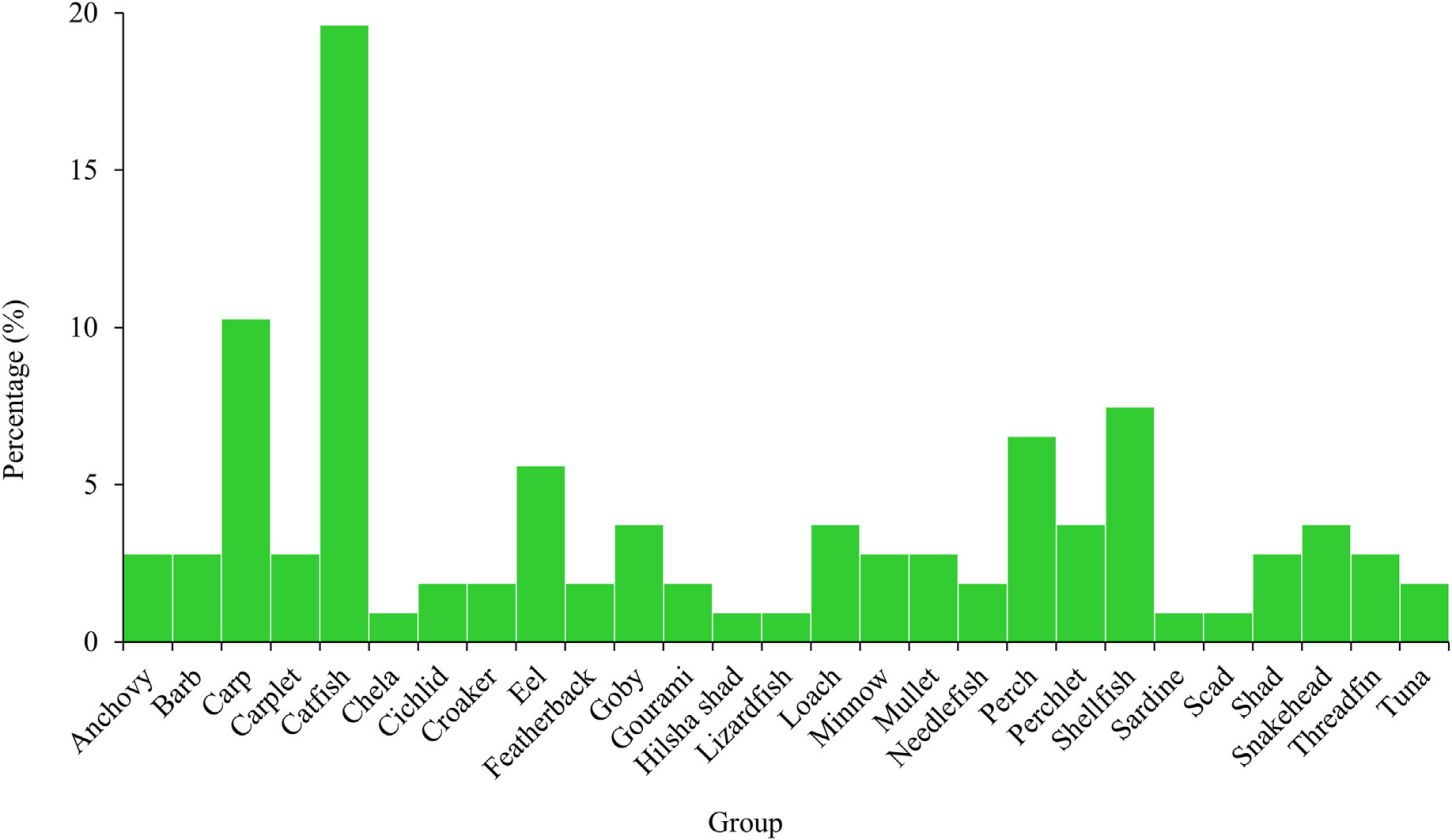
Available groups of fish and shellfishes in the Rajbari fish markets (Source: Authors Survey, 2020).

In this study, 107 species of fish and shellfish from 11 orders were documented from the markets of Rajbari Sadar (Table 2). Among them, Perciformes (28.97% of total fishes) was the most dominant orders followed by Cypriniformes (23.37%), Siluriformes (19.63%), Clupeiformes (7.48%) and Decapoda (7.48%) (Figure 3). Besides, some order of fishes was rarely found namely Anguiliformes (1.87%), Aulopiformes (0.94%), Beloniformes (1.87%), Mugliformes (2.8%), Osteoglossiformes (1.87%) and Synbranchifomes (3.73%). The most supreme group was catfish (19.63%) followed by carp (10.28%), shellfish (7.48%), perch (6.54%) and eel (5.61%) (Figure 4). Among the marine species scads and sardine were less dominating groups (0.93%). More than 50% of Perciformes fishes (i.e. *Anabas testudineas, Channa orientalis, Lepidocephalichthys guntea, Oreochromis mossambicus, Oreochromis niloticus* and so on) were commonly accessible in the marketplace (Table 2).

**Table 2:** Available fish and shellfish species in the Rajbari fish markets.

Order	Scientific name	Group	Type	Origin	Retail status	Availability	Demand	Source (District)
Anguilliformes	*Anguilla bengalensis*	Eel	FW	Capture	Single	Very rare	Low	Rajbari
	*Pisodonophis boro*	Eel	FW	Capture	Mixture	Common	Low	Rajbari
Aulopiformes	*Harpadon nehereus*	Lizard fish	MW	Capture	Single	Few	Medium	Chattogram, Cox's Bazar
Beloniformes	*Dermogenys pusillus*	Needle fish	FW	Capture	Mixture	Few	High	Rajbari
	*Xenentodon cancila*	Needle fish	FW	Capture	Mixture	Common	High	Rajbari
Clupeiformes	*Corica soborna*	Shad	FW	Capture	Single	Rare	High	Rajbari
	*Gudusia chapra*	Shad	FW	Capture	Single	Common	High	Rajbari
	*Sardinella fimbriata*	Sardine	MW	Capture	Single	Few	Medium	Chattogram, Cox's Bazar
	*Setipinna phasa*	Anchovy	FW	Capture	Single	Few	Medium	Rajbari
	*Setipinna taty*	Anchovy	FW	Capture	Single	Few	Medium	Rajbari
	*Tenualosa ilisha*	Hilsa shad	FW	Capture	Single	Common	High	Rajbari, Barishal, Chandpur
	*Tenualosa toli*	Shad	MW	Capture	Single	Few	High	Chandpur, Bhola
	*Thryssa spinidens*	Anchovy	MW	Capture	Single	Few	Low	Chattogram, Cox's Bazar
Cypriniformes	*Aristicthys nobitis*	Carp	FW ∗	Culture	Mixture	Common	High	Rajbari
	*Amblypharyngodon microlepis*	Carplet	FW	Capture	Mixture	Common	High	Rajbari
	*Amblypharyngodon mola*	Carplet	FW	Capture	Mixture	Common	High	Rajbari
	*Aspidoparia morar*	Carplet	FW	Capture	Both	Common	Medium	Rajbari
	*Banbonymus gonionotus*	Carp	FW ∗	Culture	Single	Common	Medium	Rajbari
	*Botia dario*	Loach	FW	Capture	Single	Few	High	Rajbari
	*Botia rostrata*	Loach	FW	Capture	Single	Very rare	High	Rajbari
	*Botia lohachata*	Loach	FW	Capture	Single	Very rare	High	Rajbari
	*Catla catla*	Carp	FW	Both	Both	Common	High	Rajbari
	*Chela atpar*	Chela	FW	Capture	Single	Common	High	Rajbari
	*Cirrhinus cirrhosus*	Carp	FW	Both	Both	Common	Medium	Rajbari
	*Ctenopharyngodon idella*	Carp	FW ∗	Culture	Single	Common	Low	Rajbari
	*Cyprinus carpio*	Carp	FW ∗	Culture	Single	Common	High	Rajbari
	*Hypophthalmichthys molitrix*	Carp	FW ∗	Culture	Single	Common	High	Rajbari
	*Labeo angra*	Carp	FW	Capture	Single	Very rare	High	Rajbari
	*Labeo bata*	Carp	FW	Both	Single	Common	High	Rajbari
	*Labeo calbasu*	Carp	FW	Both	Single	Common	High	Rajbari
	*Labeo gonius*	Carp	FW	Both	Single	Very rare	High	Rajbari
	*Labeo rohita*	Carp	FW	Both	Single	Common	High	Rajbari
	*Osteobrama cotio*	Minnow	FW	Capture	Single	Common	High	Rajbari
	*Pethia ticto*	Barb	FW	Both	Mixture	Common	Medium	Rajbari
	*Puntius sophore*	Barb	FW	Both	Mixture	Common	Medium	Rajbari
	*Salmostoma bacaila*	Minnow	FW	Capture	Mixture	Common	Medium	Rajbari
	*Salmostoma phulo*	Minnow	FW	Capture	Mixture	Common	Medium	Rajbari
	*Systomus sarana*	Barb	FW	Both	Mixture	Common	Medium	Rajbari
Decapoda	*Macrobrachium rosenbergii*	Prawn	FW	Both	Single	Few	High	Rajbari, Khulna
	*Macrobrachium villosimanus*	Prawn	FW	Capture	Single	Common	High	Rajbari
	*Macrobrachium lamarrei*	Prawn	FW	Capture	Both	Common	High	Rajbari
	*Macrobrachium dayanus*	Prawn	FW	Capture	Both	Common	High	Rajbari
	*Macrobrachium malcolmsonii*	Prawn	FW	Capture	Both	Common	High	Rajbari
	*Macrobrachium rude*	Prawn	FW	Capture	Both	Common	High	Rajbari
	*Metapenaeus lysianassa*	Shrimp	MW	Capture	Single	Few	High	Khulna
	*Penaeus monodon*	Shrimp	MW	Culture	Single	Few	High	Khulna, Jashore
Mugliformes	*Liza parsia*	Mullet	MW	Capture	Single	Very rare	Medium	Khulna
	*Mugil cephalus*	Mullet	MW	Capture	Single	Few	Medium	Khulna
	*Rhinomugil corsula*	Mullet	MW	Both	Single	Common	Medium	Khulna
Osteoglossiformes	*Chitala chitala*	Featherback	FW	Capture	Single	Rare	High	Rajbari
	*Notopterus notopterus*	Featherback	FW	Capture	Single	Few	High	Rajbari
Perciformes	*Anabas testudineas*	Perch	FW	Both	Single	Common	High	Rajbari, Jashore
	*Apocryptes bata*	Goby	FW	Capture	Mixture	Few	Medium	Rajbari
	*Badis badis*	Perch	FW	Capture	Mixture	Very rare	Medium	Rajbari
	*Chanda nama*	Perchlet	FW	Capture	Mixture	Rare	High	Rajbari
	*Channa marulius*	Snakehead	FW	Capture	Mixture	Very rare	Medium	Rajbari
	*Channa orientalis*	Snakehead	FW	Capture	Single	Common	Medium	Rajbari
	*Channa punctatus*	Snakehead	FW	Both	Single	Common	Medium	Rajbari
	*Channa striatus*	Snakehead	FW	Both	Single	Common	Medium	Rajbari
	*Eleutheronema tetradactylum*	Threadfin	MW	Capture	Single	Few	Medium	Chattogram, Cox's Bazar
	*Glossogobius giuris*	Goby	FW	Capture	Single	Common	High	Rajbari
	*Johnius belangerii*	Croaker	MW	Capture	Single	Few	Medium	Chattogram, Cox's Bazar
	*Katsuwonus pelamis*	Tuna	MW	Capture	Single	Very rare	Low	Chattogram, Coxs Bazar
	*Lates calcarifer*	Perch	MW	Capture	Mixture	Common	High	Khulna
	*Lepidocephalichthys annandalei*	Goby	FW	Capture	Mixture	Rare	High	Rajbari
	*Lepidocephalichthys guntea*	Goby	FW	Capture	Mixture	Common	High	Rajbari
	*Megalaspis cordyla*	Scad	MW	Capture	Both	Rare	Medium	Chattogram, Cox's Bazar
	*Nandus nandus*	Perch	FW	Capture	Single	Few	Medium	Rajbari
	*Oreochromis mossambicus*	Cichlid	FW ∗	Culture	Single	Common	Medium	Rajbari, Jashore
	*Oreochromis niloticus*	Cichlid	FW ∗	Culture	Single	Common	Medium	Rajbari, Jashore
	*Otolithoides pama*	Croaker	FW	Capture	Mixture	Common	Medium	Rajbari
	*Pampus chinensis*	Perch	MW	Capture	Mixture	Common	High	Chattogram, Cox's Bazar
	*Pampus argenteus*	Perch	MW	Capture	Single	Few	High	Chattogram, Cox's Bazar
	*Polynemus indicus*	Threadfin	MW	Capture	Single	Few	Medium	Khulna
	*Polynemous paradiseus*	Threadfin	FW	Capture	Mixture	Common	Medium	Rajbari
	*Pomadasys hasta*	Perch	MW	Capture	Single	Rare	Medium	Khulna
	*Pseudambassis baculis*	Perchlet	FW	Capture	Mixture	Common	High	Rajbari
	*Pseudambassis lala*	Perchlet	FW	Capture	Single	Common	High	Rajbari
	*Pseudambassis ranga*	Perchlet	FW	Capture	Single	Common	High	Rajbari
	*Thunnus albacores*	Tuna	MW	Capture	Single	Few	Low	Chattogram, Cox's Bazar
	*Trichogaster fasciata*	Gourami	FW	Capture	Single	Common	Medium	Rajbari
	*Trichogaster lalius*	Gourami	FW	Capture	Single	Very rare	Medium	Rajbari
Siluriformes	*Ailia coila*	Catfish	FW	Capture	Single	Common	High	Rajbari
	*Bagarius bagarius*	Catfish	FW	Capture	Both	Rare	High	Rajbari
	*Clarias batrachus*	Catfish	FW	Both	Both	Common	High	Rajbari
	*Clupisoma garua*	Catfish	FW	Capture	Both	Rare	Medium	Rajbari
	*Eutropiichthys vacha*	Catfish	FW	Capture	Single	Common	Medium	Rajbari
	*Gagata cenia*	Catfish	FW	Capture	Single	Very rare	Low	Rajbari
	*Glyptothorax telchitta*	Catfish	FW	Capture	Single	Rare	Low	Rajbari
	*Heteropneustes fossilis*	Catfish	FW	Both	Single	Common	High	Rajbari
	*Mystus bleekeri*	Catfish	FW	Both	Single	Few	High	Rajbari
	*Mystus tengara*	Catfish	FW	Both	Single	Few	High	Rajbari
	*Mystus vittatus*	Catfish	FW	Both	Single	Common	High	Rajbari
	*Ompok pabda*	Catfish	FW	Both	Single	Rare	High	Rajbari
	*Ompok bimaculatus*	Catfish	FW	Capture	Both	Very rare	High	Rajbari
	*Pangasius pangasius*	Catfish	FW	Capture	Both	Common	High	Rajbari
	*Pangasianodon hypophthalmus*	Catfish	FW ∗	Culture	Both	Common	High	Rajbari, Mymensingh, Kushtia
	*Pseudeutropius atherinoides*	Catfish	FW	Capture	Mixture	Common	Low	Rajbari
	*Rita rita*	Catfish	FW	Capture	Single	Very rare	High	Rajbari
	*Silonia silondia*	Catfish	FW	Capture	Single	Few	Low	Rajbari
	*Sperata aor*	Catfish	FW	Capture	Single	Common	High	Rajbari
	*Sperata seenghala*	Catfish	FW	Capture	Mixture	Few	High	Rajbari
	*Wallago attu*	Catfish	FW	Both	Mixture	Very rare	High	Rajbari
Synbranchiformes	*Macrognathus aculeatus*	Eel	FW	Capture	Both	Common	Low	Rajbari
	*Macrognathus pancalus*	Eel	FW	Capture	Both	Common	Low	Rajbari
	*Mastacembelus armatus*	Eel	FW	Capture	Both	Common	Low	Rajbari
	*Monopterus cuchia*	Eel	FW	Capture	Both	Rare	Low	Rajbari, Mymensingh

Note: ∗ indicates exotic species; FW means freshwater; and MW means marine water species.

Source: Authors survey, 2020

**Figure 5:**
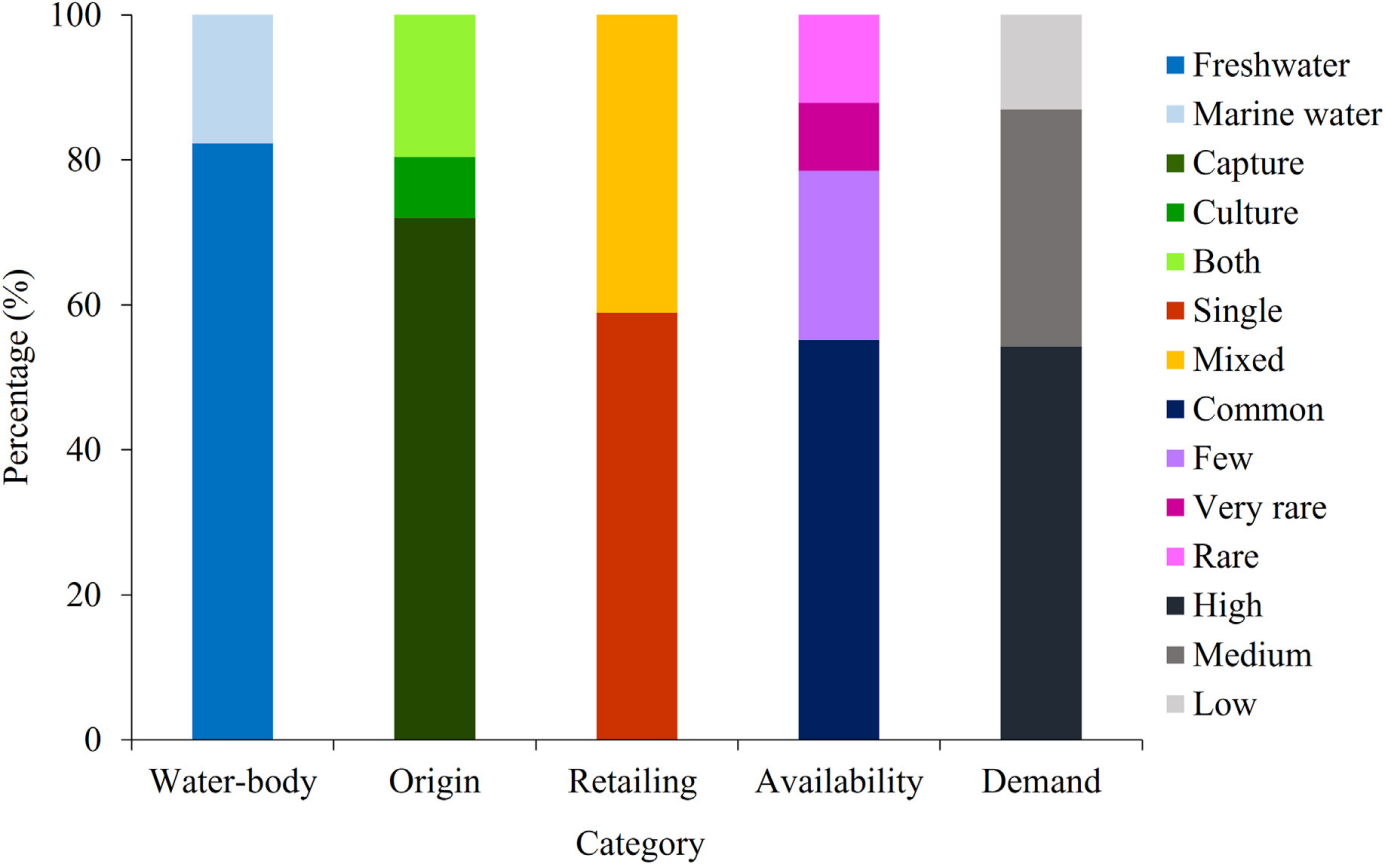
Percentage of fish and shellfish species based on water-body, origin, retailing status, availability in the market and consumers' demand (Source: Authors Survey, 2020).

The study showed that, 82.24% species were freshwater origin and dominating portion was from capture fisheries (71.96%), led by the order Cypriniformes as a single contributor for both capture and culture (Figure 5). Whereas, 8.41% was culture species arriving from both nearby farms and other districts like Jashore, Mymensingh and Kushtia. However, 55.21% of total fish and shellfish was categorized as common, whereas 23.36%, 12.15% and 9.35% of total fishes were in the few, rare and very rare categories, respectively (Figure 5). In the retail market, most of the species were being sold individually (55.88%) and small sized fishes were mostly sold in mixture based customers preference (Figure 5 and Table 2). The study also represented that, demand of most of the fishes (54.21%) were high and 13.08% fishes were of low demand including *Anguilla bengalensis*, *Pisodonophis boro*, *Gagata cenia*, *Ctenopharyngodon idella*, *Glyptothorax telchitta*, *Thryssa spinidens*, and *Thunnus albacores*. Both wild and cultivated Indian major carps were recorded from the study (Table 2). Majority exotic varieties of fishes like *Aristicthys nobitis, Banbonymus gonionotus, Ctenopharyngodon idella*, and so on were also cultivated in Rajbari district, whereas the range of its consumer demand varies from medium to high. In addition, all the recorded shellfishes including freshwater prawn and saltwater shrimp were highly demanded.

### 3.2 Available processed fish and shellfish products

**Figure 6:**
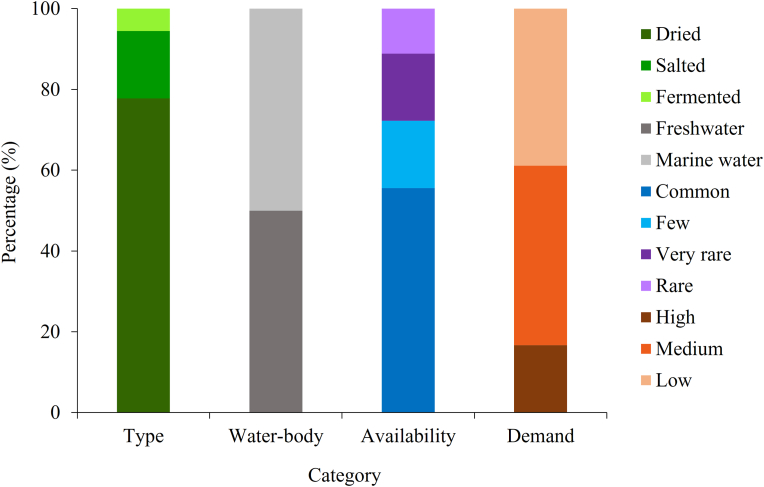
Percentage of processed fish and shellfish species based on type of product, water-body, availability in the market and consumers' demand (Source: Authors Survey, 2020).

Three types of processed fish products such as dried (77.77%), salted (16.67%) and fermented products (5.56%) were recorded from this study (Figure 6). Among the products, more than 50% (dried silver hatchet chela, dried Bombey duck, dried scaly hairfin anchovy, dried paradise threadfin, dried small-head hairtail, dried Chinese silver pomfret, dried Indo-Pacific king mackerel, dried small shrimp, salted hilsa shad, salted toil shad and salted hilsa egg) were originated from marine fishes and arrived from other cities like Dhaka and Chattogram. Demand for most of the (44.4%) products were medium and 16.67% products were highly demanded including dried Bombey duck, dried small shrimp, salted hilsa shad (Table 3 and Figure 6). In contrast, 5 processed products namely dried Ganges river sprat, dried tengara catfish, dried Indian river shad, dried mola carplet, dried small freshwater prawn and fermented two-spot barb from freshwater, were commonly available but their demand ranged from low to medium.

**Table 3:** Available of processed fish and shellfish in the Rajbari fish markets.

Scientific name	English name	Local name	Water body	Availability	Demand	Source (District)
*Corica soborna*	Dried Ganges river sprat	Kachki shutki	FW	Common	Medium	Dhaka, Chattogram
*Mystus sp.*	Dried tengara catfish	Tengra sutki	FW	Common	Medium	Dhaka
*Gudusia chapra*	Dried Indian river shad	Chapila shutki	FW	Common	Medium	Dhaka, Chattogram
*Chela atpar*	Dried silver hatchet chela	Chela shutki	MW	Common	Medium	Dhaka, Chattogram
*Amblypharyngodon mola*	Dried mola carplet	Mola shutki	FW	Common	Medium	Dhaka
*Harpadon nehereus*	Dried Bombey duck	Loittya shutki	MW	Common	High	Dhaka
*Channa punctatus*	Dried spotted snakehead	Taki sutki	FW	Rare	Low	Dhaka
*Setipinna taty*	Dried scaly hairfin anchovy	Phasa shutki	MW	Rare	Low	Dhaka, Chattogram
*Polynemus paradiseus*	Dried paradise threadfin	Taposi shutki	MW	Rare	Low	Dhaka, Chattogram
*Eupleurogrammus muticus*	Dried smallhead hairtail	Churi shutki	MW	Few	Low	Dhaka, Chattogram
*Pampus chinensis*	Dried Chinese silver pomfret	Rup chanda shutki	MW	Very rare	Low	Dhaka, Chattogram
*Scomberomorus guttatus*	Dried Indo-Pacific king mackerel	Maitta sutki	MW	Very rare	Low	Dhaka
*Macrobrachium sp.*	Dried small freshwater prawn	Torkari chingri shutki	FW	Common	Medium	Dhaka, Sylhet
*Acetes sp.*	Dried small shrimp	Gura chingri shutki	MW	Common	High	Dhaka, Sylhet
*Puntius ticto*	Fermented two-spot barb	Chepa shutki	FW	Few	Low	Dhaka, Mymensingh
*Tenualosa ilisha*	Salted hilsa shad	Nona ilish	MW	Common	High	Dhaka
*Tenualosa ilisha*	Salted hilsa egg	Nona ilish dim	MW	Few	Medium	Dhaka
*Tenualosa toli*	Salted toli shad	Nona chandina	MW	Common	Medium	Dhaka

Note: FW means freshwater and MW means marine water species.

Source: Authors survey, 2020

### 3.3 Marketing channel and value chain

**Figure 7:**
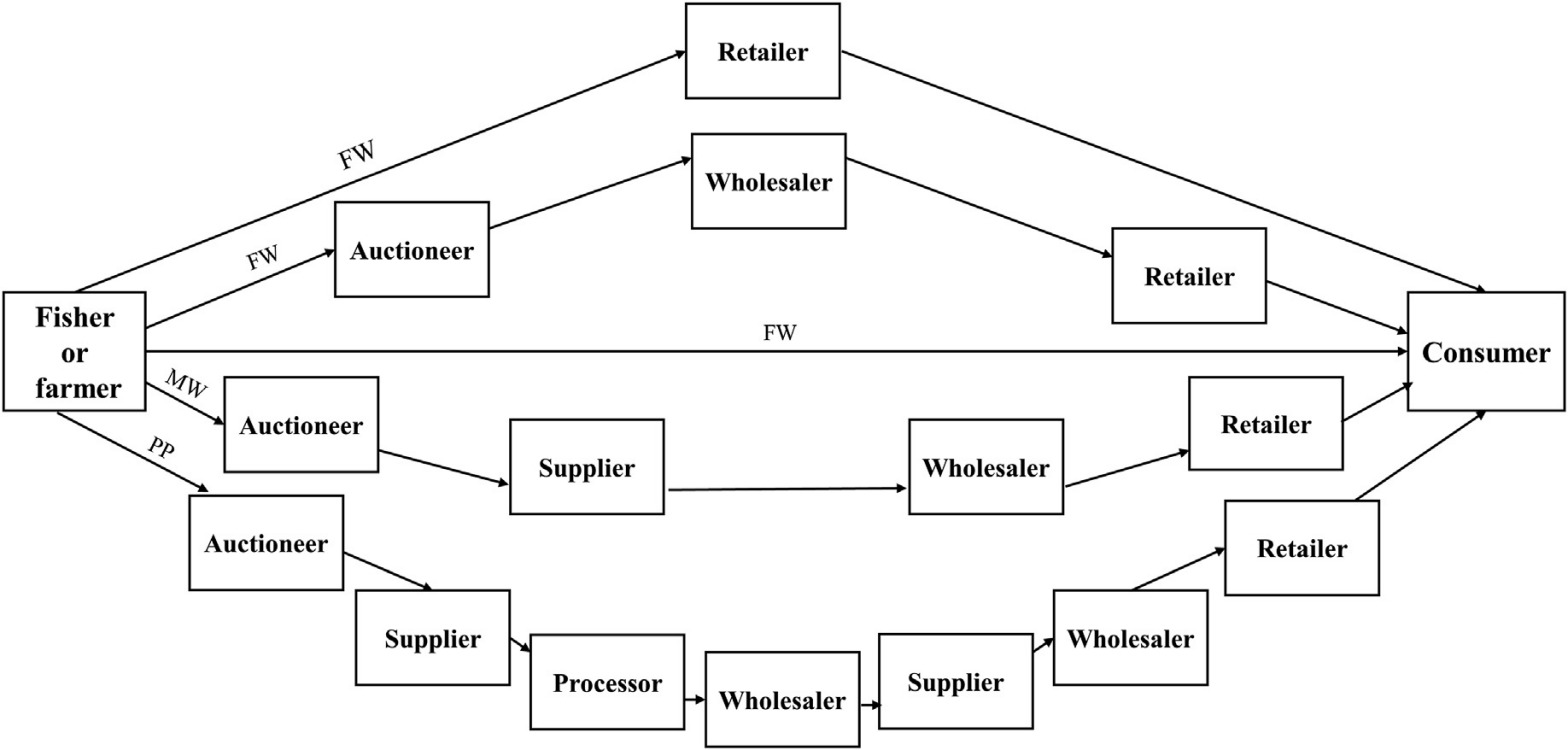
Marketing channel in the Rajbari fish markets (Here, FW: Freshwater fish, MW: Marine fish and PP: Processed fish and shellfish product) (Source: Authors Survey, 2020).

Basically, the length of marketing channel depends on the numbers of intermediaries involved with it. Findings from the observed area are that, there are three, four and seven intermediaries involved in the marketing channel of freshwater, marine and processed fishes and shellfishes, respectively though it may vary in term of individual transaction (Figure 7). Usually, auctioneers facilitate the transaction in between fishermen or farmers and wholesalers or suppliers through a shouting auction. Then in case of freshwater it reaches to the consumers through retailers. On the other hand, it moves to the ultimate consumers through wholesalers and retailers in case of marine fish. Sometimes consumers get freshwater fish directly from fishermen or farmers or through retailers. Whereas, for processed product after getting it from the fishermen through auctioneers suppliers sale it to processors. After completing processing, processors handover it to suppliers through wholesalers and consumers of Rajbari get it through wholesaler and retailers from those suppliers.

**Figure 8:**
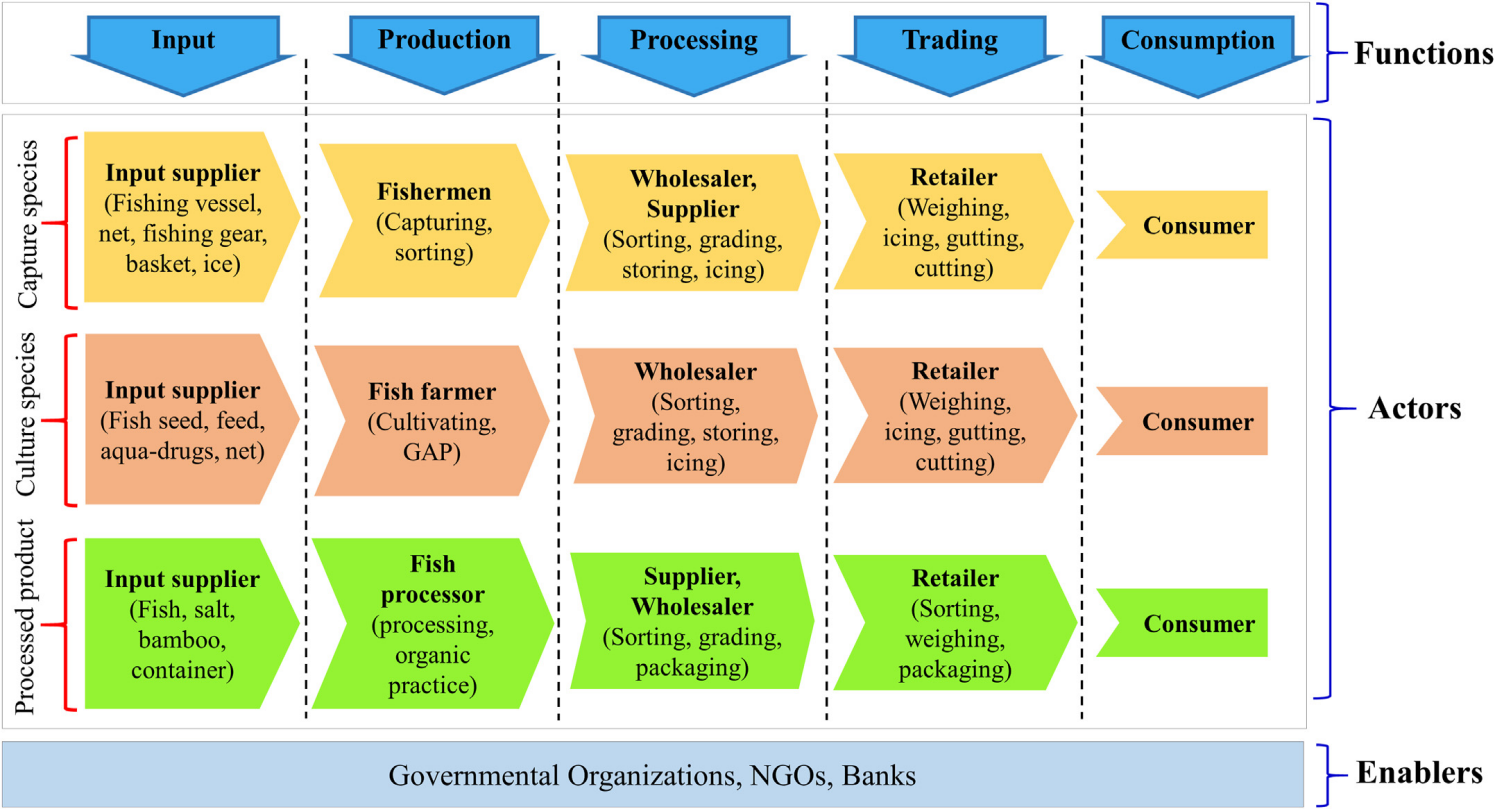
Map showing the value chain of fish and shellfish of Rajbari markets. Here, GAP means good aquaculture practice (Source: Authors Survey, 2020).

Fish markets of Rajbari include different value chain actors like input suppliers, fishermen, fish farmers, fish processors, suppliers, wholesalers, retailers and ultimate consumer in its value chain (Figure 8). Input suppliers provide net, boat, basket, fingerling, fish feed, aqua-medicine, salt, processing container and so on for fishermen, farmers and processors. In production level they perform different values adding activities like capturing, culturing, sorting trash species, practicing good aquaculture and producing organic fishery products. Then wholesalers and suppliers perform sorting, grading, storing, icing and packaging for improving the quality and shelf life of products and ensuring smoother distribution. Finally, retailers practice value enhancing activities like weighing, gutting, cutting and packaging before selling it to the ultimate consumer. Throughout the different level of functions different facilitators including Governmental Organizations, NGOs and financial organizations help value chain actors in term of technical and financial aspects.

### 3.4 Fish price and market margin

**Figure 9:**
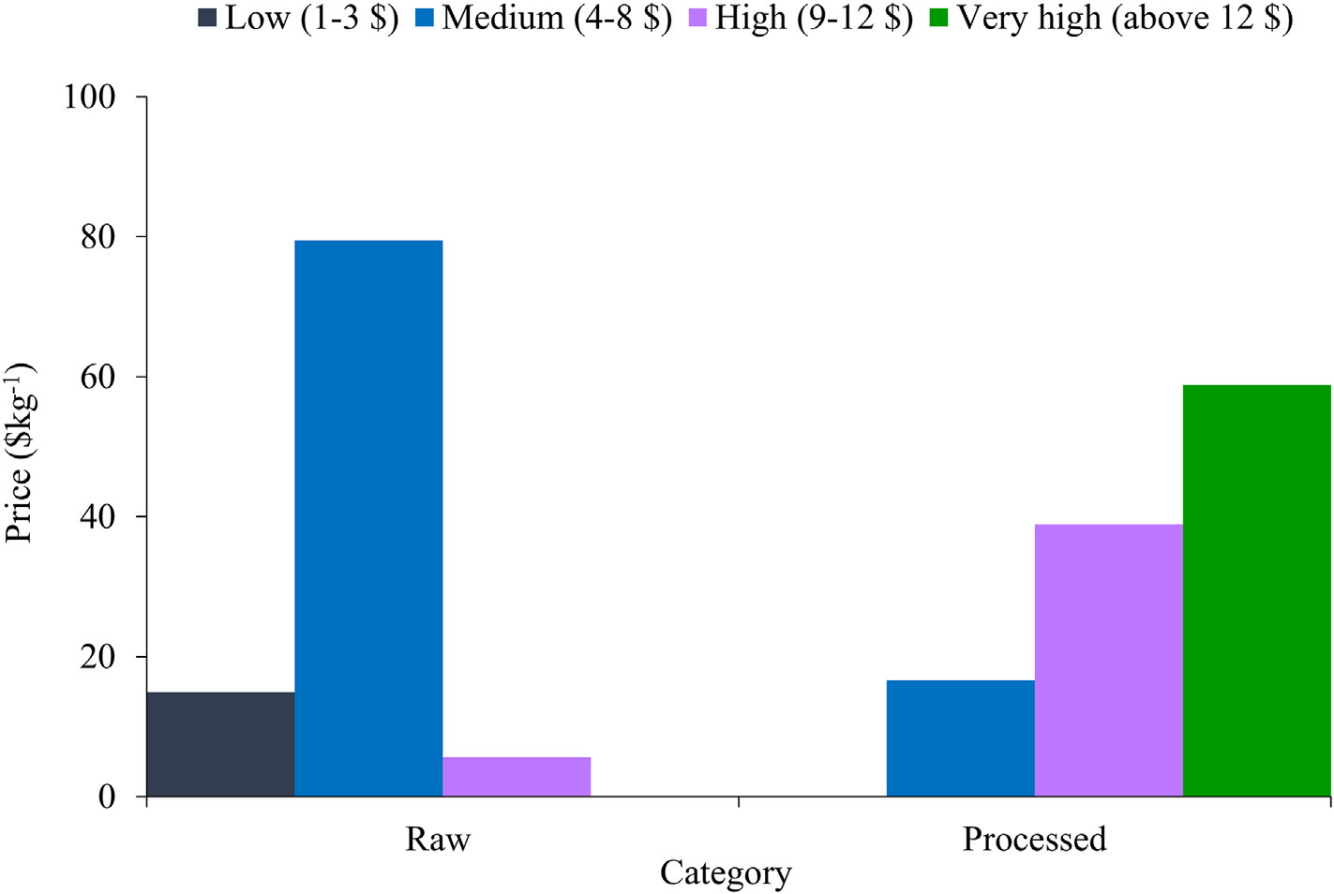
Retail price ($ kg^−1^) of raw fish, shellfish and processed products in Rajbari fish markets (Source: Authors Survey, 2020).

Fish, shellfish and processed products price gradually increased through the passage from producer to consumer level (Tables 4, 5, and 6). Retail price of most of the raw fishes (79.44%) including freshwater and marine species was medium and it didn't exceed $12 per kg; whereas no processed product was found selling at low-price (Figure 9). Like shrimp, the price of marine fishes was comparatively higher than those from local origin. Tuna (*Thunnus albacores*) as an example that mainly comes from Chattogram and Cox's Bazar, its price varies from $6 to 9.60 kg^−1^. The prices of *Tenualosa ilisha* and *Tenualosa toli* from Rajbari, Barishal, Chandpur and Bhola varied from $7.20 to 10.80 kg^−1^ (Tables 4 and 5). *Penaeus monodon* was recorded as high valued ($12 kg^−1^) saltwater shellfish (Table 5). Contrarily, in the retail market price of *Harpadon nehereus* and *Sardinella fimbriata* ($1.9 kg^−1^) was lowest. Majority (58.88%) processed fishes were found in very high value group and only 16.67% products were available at medium price. The price range of freshwater dried fish was from $6 to 12 kg^−1^ in wholesale market and from $8.40 to 14.40 kg^−1^ in retail market (Table 6).

**Table 4:** Freshwater fish and shellfish price and market margin at different stages of market channel.

Scientific name	Price ($ kg^−1^)				Market margin (%)		
	PR	AC	WH	RT	AC	WH	RT
*Ailia coila*	4.7	5.0	6.0	8.4	6.98	16.65	28.57
*Amblypharyngodon microlepis*	2.3	2.7	4.2	5.4	12.90	36.31	22.22
*Amblypharyngodon mola*	2.3	2.7	4.2	5.4	12.90	36.31	22.22
*Anabas testudineas*	2.3	2.7	3.6	4.8	12.90	25.69	25.00
*Anguilla bengalensis*	5.0	5.6	6.0	8.4	10.37	7.00	28.57
*Apocryptes bata*	1.4	1.6	2.4	4.8	14.02	32.15	50.00
*Aristicthys nobitis*	1.1	1.3	1.8	3.0	17.93	28.92	40.00
*Aspidoparia morar*	2.6	2.9	5.4	6.6	12.07	46.11	18.18
*Badis badis*	3.5	3.8	4.8	6.0	9.09	20.04	20.00
*Bagarius bagarius*	4.1	4.4	6.0	8.4	7.89	26.34	28.57
*Banbonymus gonionotus*	2.1	2.3	3.3	3.6	10.00	28.57	9.54
*Botia dario*	3.5	3.7	4.8	6.0	6.25	21.95	20.52
*Botia lohachata*	3.5	3.7	4.8	6.0	6.25	21.95	20.52
*Botia rostrata*	3.5	3.7	4.8	6.0	6.25	21.95	20.52
*Catla catla*	1.4	1.6	2.4	3.6	14.02	32.15	33.33
*Chanda nama*	0.9	1.2	1.8	3.0	19.83	35.56	40.00
*Channa marulius*	2.9	3.3	4.2	5.4	10.81	22.38	22.22
*Channa orientalis*	2.3	2.6	3.6	4.8	8.94	28.92	25.00
*Channa punctatus*	1.4	1.7	3.0	4.2	19.54	42.00	28.57
*Channa striatus*	3.5	3.8	4.8	6.0	9.07	20.04	20.00
*Chela atpar*	2.1	2.3	3.6	4.8	10.15	35.39	25.00
*Chitala chitala*	5.2	5.6	6.0	7.2	6.25	6.96	16.67
*Cirrhinus cirrhosus*	0.9	1.2	1.8	3.0	19.83	35.56	40.00
*Clarias batrachus*	4.1	4.4	6.0	7.2	7.91	26.34	16.67
*Clupisoma garua*	4.1	4.4	4.8	6.0	7.91	7.92	20.00
*Corica soborna*	1.7	1.9	3.0	4.2	6.25	37.97	28.57
*Ctenopharyngodon idella*	0.9	1.2	1.8	3.0	19.83	35.56	40.00
*Cyprinus carpio*	0.9	1.2	1.8	3.0	19.83	35.56	40.00
*Dermogenys pusillus*	2.9	3.1	3.6	4.8	7.40	12.78	25.00
*Eutropiichthys vacha*	4.4	4.7	6.0	7.2	4.95	22.50	16.67
*Gagata cenia*	4.4	4.7	6.0	7.2	4.95	22.50	16.67
*Glossogobius giuris*	2.6	3.3	4.2	6.6	21.47	22.38	36.36
*Glyptothorax telchitta*	4.7	5.0	6.0	8.4	6.98	16.65	28.57
*Gudusia chapra*	2.6	3.3	3.6	4.8	21.47	9.44	25.00
*Heteropneustes fossilis*	4.4	4.7	6.0	7.2	4.95	22.50	16.67
*Hypophthalmichthys molitrix*	0.9	1.2	1.8	3.0	19.83	35.56	40.00
*Labeo angra*	2.7	2.9	3.6	4.8	8.07	19.17	25.00
*Labeo bata*	1.2	1.3	1.8	3.0	9.09	28.92	40.00
*Labeo calbasu*	2.1	2.3	3.0	5.4	10.00	22.46	44.44
*Labeo gonius*	2.6	2.9	3.6	4.8	12.07	19.17	25.00
*Labeo rohita*	1.9	2.1	3.0	5.4	10.96	30.33	44.44
*Lepidocephalichthys annandalei*	3.0	3.3	4.2	5.4	7.24	22.38	22.22
*Lepidocephalichthys guntea*	2.7	2.9	3.6	4.8	8.07	19.17	25.00
*Macrobrachium dayanus*	3.3	4.1	4.8	8.4	19.90	15.21	42.86
*Macrobrachium lamarrei*	3.3	3.5	4.8	6.0	6.69	27.29	20.00
*Macrobrachium malcolmsonii*	3.7	4.1	4.8	8.4	8.56	15.21	42.86
*Macrobrachium rosenbergii*	5.2	5.8	7.2	12.0	10.07	19.17	40.00
*Macrobrachium rude*	3.8	4.1	4.8	8.4	5.70	15.21	42.86
*Macrobrachium villosimanus*	5.4	5.6	6.0	8.4	4.17	6.96	28.57
*Macrognathus aculeatus*	4.2	4.4	4.8	6.0	5.26	7.92	20.00
*Macrognathus pancalus*	4.2	4.4	5.4	6.6	5.26	18.15	18.18
*Mastacembelus armatus*	3.5	4.1	6.0	7.2	14.25	32.17	16.67
*Monopterus cuchia*	5.2	5.6	6.0	7.2	6.25	6.80	16.69
*Mystus bleekeri*	4.1	4.7	6.0	7.2	12.47	22.50	16.67
*Mystus tengara*	4.1	4.7	6.0	7.2	12.47	22.50	16.67
*Mystus vittatus*	4.1	4.7	6.0	7.2	12.47	22.50	16.67
*Nandus nandus*	1.7	2.1	3.0	4.2	16.53	30.33	28.57
*Notopterus notopterus*	1.5	1.7	2.4	3.6	13.10	27.50	33.33
*Ompok bimaculatus*	4.1	4.4	4.8	6.0	7.89	7.92	20.00
*Ompok pabda*	4.1	4.4	4.8	6.0	7.89	7.92	20.00
*Oreochromis mossambicus*	0.8	0.9	1.2	1.9	12.46	22.50	37.50
*Oreochromis niloticus*	0.8	0.9	1.2	1.9	12.46	22.50	37.50
*Osteobrama cotio*	2.9	3.3	4.2	5.4	10.71	22.46	22.22
*Otolithoides pama*	2.9	3.3	4.2	6.6	10.81	22.38	36.36
*Pangasianodon hypophthalmus*	4.2	5.8	6.0	7.2	28.00	3.08	16.67
*Pangasius pangasius*	0.9	1.2	1.8	3.0	19.83	35.56	40.00
*Pethia ticto*	0.9	1.2	1.8	3.0	20.00	35.39	40.00
*Pisodonophis boro*	1.9	2.1	3.0	4.2	11.11	30.22	28.57
*Polynemous paradiseus*	4.2	4.4	4.8	7.2	5.26	7.92	33.33
*Pseudambassis baculis*	0.9	1.0	1.8	3.0	11.15	41.85	40.00
*Pseudambassis lala*	0.9	1.0	1.8	3.0	11.15	41.85	40.00
*Pseudambassis ranga*	0.9	1.0	1.8	3.0	11.15	41.85	40.00
*Pseudeutropius atherinoides*	4.4	4.7	6.0	8.4	5.00	22.46	28.57
*Puntius sophore*	0.9	1.0	1.8	3.0	11.15	41.85	40.00
*Rita rita*	5.0	5.2	6.6	7.8	4.38	20.76	15.38
*Salmostoma bacaila*	1.5	1.7	2.4	4.8	13.10	27.50	50.00
*Salmostoma phulo*	1.2	1.4	1.8	5.4	16.92	22.22	66.67
*Setipinna phasa*	2.7	2.9	3.6	4.8	8.07	19.17	25.00
*Setipinna taty*	2.7	2.9	3.6	4.8	8.07	19.17	25.00
*Silonia silondia*	5.0	5.2	6.0	8.4	4.38	12.83	28.57
*Sperata aor*	5.0	5.2	6.0	8.4	4.38	12.83	28.57
*Sperata seenghala*	5.0	5.2	6.6	7.8	4.38	20.76	15.38
*Systomus sarana*	2.7	2.9	3.6	4.8	8.07	19.17	25.00
*Tenualosa ilisha*	5.8	6.4	7.2	10.8	9.09	11.16	33.33
*Trichogaster fasciata*	3.7	4.1	4.8	6.0	8.56	15.21	20.00
*Trichogaster lalius*	3.7	4.1	4.8	8.4	8.56	15.21	42.86
*Wallago attu*	4.9	5.2	5.8	6.5	6.60	9.83	10.77
*Xenentodon cancila*	2.9	3.1	3.6	4.8	7.40	12.78	25.00

Note: PR = Producer indicates fishermen for capture fish and fish farmer for culture species; AC = Auctioneer; WH = Wholesaler; and RT = Retailer.

Source: Authors survey, 2020

**Table 5:** Marine fish and shellfish price and market margin at different stages of market channel.

Scientific name	Price ($ kg^−1^)					Market margin (%)			
	PR	AC	SP	WH	RT	AC	SP	WH	RT
*Eleutheronema tetradactylum*	2.7	2.9	3.5	4.8	6.0	8.07	16.62	27.29	20.00
*Harpadon nehereus*	0.6	0.7	1.1	1.4	1.9	16.92	33.33	27.08	25.00
*Johnius belangerii*	2.3	2.6	2.9	3.6	6.0	8.98	12.03	19.17	40.00
*Katsuwonus pelamis*	3.7	4.1	4.7	6.0	9.6	8.56	12.47	22.50	37.50
*Lates calcarifer*	2.7	2.9	3.5	4.8	6.0	8.07	16.62	27.29	20.00
*Liza parsia*	2.7	2.9	3.5	4.8	6.0	8.07	16.62	27.29	20.00
*Megalaspis cordyla*	3.7	4.1	4.7	6.0	8.4	8.56	12.47	22.50	28.57
*Metapenaeus lysianassa*	2.7	2.9	3.5	4.8	8.4	8.07	16.62	27.29	42.86
*Mugil cephalus*	2.7	2.9	3.5	4.8	6.0	8.07	16.62	27.29	20.00
*Pampus argenteus*	3.7	4.1	4.7	6.0	9.6	8.56	12.47	22.50	37.50
*Pampus chinensis*	3.7	4.1	4.7	6.0	9.6	8.56	12.47	22.50	37.50
*Penaeus monodon*	5.2	5.8	7.0	8.4	12.0	10.14	16.62	16.90	30.00
*Polynemus indicus*	2.3	2.6	2.9	3.6	6.0	8.98	12.03	19.17	40.00
*Pomadasys hasta*	2.7	2.9	3.5	4.8	6.0	8.07	16.62	27.29	20.00
*Rhinomugil corsula*	2.3	2.6	2.9	3.6	4.8	8.98	12.03	19.17	25.00
*Sardinella fimbriata*	0.6	0.7	1.1	1.4	1.9	16.92	33.33	27.08	25.00
*Tenualosa toli*	0.7	0.9	1.2	1.8	4.2	24.73	19.83	35.56	57.14
*Thryssa spinidens*	2.7	2.9	3.5	4.8	6.0	8.07	16.62	27.29	20.00
*Thunnus albacores*	3.8	4.1	4.7	6.0	9.6	5.70	12.47	22.50	37.50

Note: PR = Producer indicates fishermen for capture fish and fish farmer for culture species; AC = Auctioneer; SP = Supplier; WH = Wholesaler; and RT = Retailer.

Source: Authors survey, 2020

**Table 6:** Processed fish and shellfish price from processing yard to retailer market and market margin.

Products	Price ($ kg^−1^)				Market margin (%)		
	PY	SP	WH	RT	SP	WH	RT
Dried Ganges river sprat	4.7	5.2	7.2	10.8	11.11	27.31	33.33
Dried tengara catfish	8.1	9.3	12.0	14.4	12.46	22.50	16.67
Dried Indian river shad	5.2	5.8	8.4	10.8	10.00	30.77	22.22
Dried silver hatchet chela	4.7	5.2	7.2	10.8	11.11	27.31	33.33
Dried mola carplet	4.1	4.7	6.0	8.4	12.50	22.46	28.57
Dried Bombey duck	4.1	4.7	6.0	8.4	12.50	22.46	28.57
Dried spotted snakehead	8.1	9.3	12.0	14.4	12.46	22.50	16.67
Dried scaly hairfin anchovy	5.8	7.0	8.4	10.8	16.67	16.92	22.22
Dried paradise threadfin	5.8	7.0	8.4	10.8	16.67	16.92	22.22
Dried smallhead hairtail	5.8	7.0	7.2	14.4	16.67	3.08	50.00
Dried Chinese silver pomfret	5.8	7.0	8.4	10.8	16.67	16.92	22.22
Dried Indo-Pacific king mackerel	8.7	10.5	14.4	18.0	16.67	27.31	20.00
Dried small freshwater prawn	8.1	9.9	12.0	18.0	17.65	17.62	33.33
Dried small shrimp	2.3	2.9	3.6	8.4	20.00	19.23	57.14
Fermented two-spot barb	5.2	6.4	8.4	9.6	18.18	23.85	12.50
Salted hilsa shad	5.2	6.4	8.4	14.4	18.18	23.85	41.67
Salted hilsa egg	8.1	9.3	12.0	19.2	12.46	22.50	37.50
Salted toli shad	5.2	5.8	8.4	14.4	10.00	30.77	41.67
Here, PY = Processing yard; SP = Supplier; WH = Wholesaler; and RT = Retailer.							

Source: Authors survey, 2020

The more intermediaries are involved in the channel the more market margin resulted for their value additional activities (Tables 4, 5, and 6). In case of freshwater fishes, higher market margin was observed in carp, cichlid, goby, loach, minnow, perchlet and shad. In marine fishes, the higher market margin was observed in croaker, hilsa, shellfish and tuna those are arrived from other cities like Cox's Bazar, Chattogram and Khulna which involves several transport stages, trader categories and intermediaries that undoubtedly lead in higher market margin (Tables 2 and 5). Among the freshwater and marine species the margin of *Salmostoma phulo* and *Tenualosa toli* in the retail market were 66.67% and 57.14%, respectively (Tables 4 and 5). On the other hand, dried small shrimp showed the dominating margin value for at the consumer level (Table 6).

### 3.5 Existing constraints in the markets

The problems associated with the fish markets reported by the participants had several dimensions and category, for instance, some were serious and tough to change while some were minor and relatively easy to mend. Using AHP method, matrix on the constraints were formed on the basis of respondents' response (Table 7). Synthesized matrix of the constraints based on Table 8 shown that the unplanned market location (Eigen-vector = 0.30) ranks first position as the most serious constraints for fish market followed by insufficient drainage system (Eigen-vector = 0.17), low supply of fish for COVID-19 pandemic (Eigen-vector = 0.13), high transportation cost (Eigen-vector = 0.12), and traditional fish transportation system (Eigen-vector = 0.08). ‘Unplanned market location’ indicates undeveloped market structure and marketplace; and as a result the traders and customers have to suffer from lacking space during transaction and unloading of the products. The study showed that, high transportation cost is one of their major problems for the species arriving from distantly regions like coastal areas or when waterways are used. As the survey was carried out during COVID-19 pandemic, the participants reported the lockdown situation as one of their sufferings. Even after lifting the transportation and movement restrictions, actors of fish value chain are still overburdened by the loss occurred due to the lockdown. However, unhygienic environment and lack of electricity are also matter of concern. Though lack of modern preservation practice and poor knowledge on post-harvest fish handling are at the bottom of the list, they are significant in other way, consumer's wellbeing.

**Table 7:** Pair-wise comparison of the constraints faced by the participants.

	A	B	C	D	E	F	G	H	I	J
A	1	3	5	5	3	7	7	7	9	5
B	1/3	1	3	3	3	3	5	5	5	5
C	1/5	1/3	1	3	3	5	3	5	3	3
D	1/5	1/3	1/3	1	5	5	3	3	5	5
E	1/3	1/3	1/3	1/5	1	3	1	3	5	3
F	1/7	1/3	1/5	1/5	1/3	1	3	3	3	5
G	1/7	1/5	1/3	1/3	1	1/3	1	1	3	3
H	1/7	1/5	1/5	1/3	1/3	1/3	1	1	5	3
I	1/9	1/5	1/3	1/5	1/5	1/3	1/3	1/5	1	3
J	1/5	1/5	1/3	1/5	1/3	1/5	1/3	1/3	1/3	1

Note: A = Unplanned market location; B= Insufficient drainage system; C = Low supply of fish for COVID-19 pandemic; D = High transportation cost; E = Traditional fish transportation system; F= Few customer due to COVID-19 pandemic; G = Unhygienic environment in market; H= Lack of continuous electricity; I= Lack of modern preservation practice; and J = Poor knowledge on post-harvest fish handling.

Source: Authors survey, 2020

**Table 8:** Synthesized matrices of constraints from the decision of participants.

	A	B	C	D	E	F	G	H	I	J	Eigen vector	Rank
A	0.36	0.49	0.45	0.37	0.17	0.28	0.28	0.25	0.23	0.14	0.30	1
B	0.12	0.16	0.27	0.22	0.17	0.12	0.20	0.18	0.13	0.14	0.17	2
C	0.07	0.05	0.09	0.22	0.17	0.20	0.12	0.18	0.08	0.08	0.13	3
D	0.07	0.05	0.03	0.07	0.29	0.20	0.12	0.11	0.13	0.14	0.12	4
E	0.12	0.05	0.03	0.01	0.06	0.12	0.04	0.11	0.13	0.08	0.08	5
F	0.05	0.05	0.02	0.01	0.02	0.04	0.12	0.11	0.08	0.14	0.06	6
G	0.05	0.03	0.03	0.02	0.06	0.01	0.04	0.04	0.08	0.08	0.04	7
H	0.05	0.03	0.02	0.02	0.02	0.01	0.04	0.04	0.13	0.08	0.04	8
I	0.04	0.03	0.03	0.01	0.01	0.01	0.01	0.01	0.03	0.08	0.03	9
J	0.07	0.03	0.03	0.01	0.02	0.01	0.01	0.01	0.01	0.03	0.02	10
Here, λ = 11.38; CI (Consistency Index) = 0.15; N = 10; RI (Random Index) = 1.49; and CR (Consistency Ratio) = 10%.												

Note: A = Unplanned market location; B= Insufficient drainage system; C = Low supply of fish for COVID-19 pandemic; D = High transportation cost; E = Traditional fish transportation system; F= Few customer due to COVID-19 pandemic; G = Unhygienic environment in market; H= Lack of continuous electricity; I= Lack of modern preservation practice; and J = Poor knowledge on post-harvest fish handling.

Source: Processed results.

## 4 Discussion

The markets of Rajbari are consisted of unplanned structure and a number of traders dealing with raw and processed fish shellfishes. The sources of freshwater capture fisheries in Rajbari are mostly the River Padma, Gorai-the tributary of the Padma, vast floodplain areas during the rainy season and culture ponds (BBS, 2011; Nadia et al., 2021). Joadder et al. (2015) and Hanif et al. (2016) reported that, the biodiversity of fishes in the Padma River and the Gorai River is high including small indigenous fish species, Indian major carps, catfishes and so on. Consequently, the number of available species also outnumbered other previous studies on fish markets in different regions of Bangladesh (Roy et al., 2020a; Rahman and Islam, 2020). Considerable amount of saltwater species and processed fish and shellfish are also listed during study, which indicates occasional demand of the inhabitants for the products. But the collection of marine stock in the survey area was lower than the markets of Southern Bangladesh. Note that, the availability of fishes in the market also depends on several factors like geographical location, water bodies, season, transportation, price, nutrition and storage facilities (Ahmed et al., 2005, 2012). From the study of Rahman and Islam (2020), it was revealed that, rohu, pangas, tilapia and hilsa were the most frequently consumed fish in Rangpur city of Bangladesh which is in accordance with the present study. It might be due to the affordable price of these fish and taste of hilsa throughout the Bangladesh. In addition, the eel varieties of fishes (*Anguilla bengalensis* and *Pisodonophis boro*) were available in the Rajbari fish market, but the consumer demand for these fish was low which might be due to fish morphology alike snake. However, fish supply is also influenced by the biological environment, the technology used, the policy and institutional environment, and the producer's profile, and fish demand is influenced by policy and profile of consumers (Das et al., 2020; Rahman and Islam, 2020).

In primary market, fishermen brought capture fishes to the nearby fish landing center and suppliers purchased those fishes and sell their consignment to the retailers through auctioneers or wholesalers. Approximately 70% of total fishes in the landing center were handled by supplier who had to pay a significant amount of commission to the wholesalers for selling the products (Rabbani et al., 2017). Composition of the marketing channels found from the present study is similar to several previous studies (Gowsalya et al., 2019; Grema et al., 2020; Roy et al., 2020a; Mebrate and Worku, 2020; Uba et al., 2020). It is needless to say that fishes from the nearby water resources have to pass shorter distribution channel, their price in wholesale and retailer are comparatively lower; however, their consumer demand is high in the study area. It might be due to more freshness of the fishes which acts as a vital determinant controlling the consumption level (Rahman and Islam, 2020; Baset, 2020; Das et al., 2020).

Some studies documented an inverse relationship between supply of fish and market price (Hasan and Middendorp, 1999; Briones et al., 2004; Burger et al., 2004; Dey et al., 2008) in Bangladeshi market, for example, increased supply of certain fish could reduce market prices by 5–16% (Dey, 2000). The price of freshwater prawn *Macrobrachium rosenbergii* and *Macrobrachium villosimanus* are higher than most of the freshwater fish excluding hilsa at the producer level (Table 4). It might be due to higher production cost for the prawn farmers and comparatively lower harvest than the other shellfishes. On the other hand, lowest price of freshwater *Chanda nama*, *Cirrhinus cirrhosis*, *Ctenopharyngodon idella*, *Cyprinus carpio*, *Hypophthalmichthys molitrix*, *Pangasius pangasius*, *Pethia ticto*, *Pseudambassis baculis*, *Pseudambassis lala* and *Pseudambassis ranga* was $ 0.9 kg^−1^ at the producer stage. Higher production and harvest from the aquafarms and seasonal water bodies are major reasons behind this low price. Hilsa price varies across seasons and according to a survey in southern districts (Bhola and Barishal) and Dhaka, the average price of hilsa throughout the year was around $8 kg^−1^ but the average price during ban season was around $7.5 kg^−1^ at entry point and $9 kg^−1^ at sales point (Porras et al., 2017). Whereas, in the present study, hilsa price at fishermen level was lower ($5.8 kg^−1^) than the previous study due to the location of Rajbari being adjacent to the River Padma and demand for this riverine hilsa is higher than the saltwater origin. A study in the retail markets of Khulna revealed that, price of marine species ranged from $2.3 kg^−1^ to $7 kg^−1^ which is much lower than the present study (Roy et al., 2020a). Das et al. (2020) reported that, combined effect of several factor like species, district or origin, traders and weight of fish significantly influence their pricing. In the landing center or the wholesale market, the traders predominantly took care of landing, handling, and post-landing tasks including cleaning, sorting, grading and icing of fishes (Ahmed et al., 2012). The price of marine fishes depends on several factors such as distance to onshore, distance to the market, number of buyers at onshore, market channel, season, transport cost per vessel (Sambuo et al., 2021). In Bangladesh, there are 7 major wholesale markets of processed fish products and from those wholesale markets, most of the products are supplied throughout the country (Hossain et al., 2013). Note that, the price of the processed fish was comparatively higher than that of the raw fish depending on species, size of species, and quality of fishes, processing method, labor cost, long marketing channel, transportation and seasons. According to Islam et al. (2006), the factory owners of drying fish pay commission to the suppliers when they sell fish to the wholesalers in their storehouse locally known as arat that accounts about 12% of total marketing cost. Consecutively, Amin et al. (2012) also mentioned that 70% of processed fish was bought by wholesalers, followed by 22%, 6% and 2% of total processed fish bought by suppliers, retailers and consumers, respectively from the Kutubdia Island which is recognized as a fish processing area in Bangladesh. In contrast to frozen fish marketing, intermediaries involved in dry fish marketing incur more costs since the fish to be marketed are dried up and processed to sell it ensuring salubrious condition (Islam et al., 2006). Though the inhabitants of the study area used to consume fresh fishes, they also prefer processed fish products depending on the special occasion, tradition and income instead of the higher price per unit (Rahman and Islam, 2020).

In the study, higher margin of *Salmostoma phulo* and *Tenualosa toli* resulted due to their local origin from river, low price at fishermen or producers phase and higher demand (Tables 4 and 5). Moreover, small dried shrimps are also low as most of the stocks are being captured as trash species which results in low production cost, but through the market channel the value is enhanced comparatively higher than the other processed products for high demand. Though price of dried tengara catfish at the processing yard was higher ($8.1 kg^−1^) than other products, margin at retailer phase (16.67%) was comparatively lower and it might be due to lower consumer demand (Table 6). Ahmed (2007) observed the highest average market margin per kilogram of hilsa in secondary market followed by retail and primary markets which is similar to the present study. Since the availability and demand of carp, cichlid and goby were common and high, respectively, their market margin was high. The present study showed that, as the product move forward through the intermediaries the price uplifted resulting higher margin for their value additional activities. A study conducted on fish trade in Istanbul, Turkey revealed that, rent, employee fees, electricity-water, ice, transportation, taxes, municipal tax, commission fee, and value added tax enhance the fish price when it pass from fishers to traders (Kaygisiz and Eken, 2018).

There were a number of problems associated with the fish markets reported by sellers and customers those were somewhat in agreement with the longstanding problems of almost all of the fish markets in Bangladesh except pandemic effect (Kaygisiz and Eken, 2018; Roy et al., 2020a; Kamaylo et al., 2021). Unplanned market structure arise uneven marketing system like increase of temporary retailers in the markets, space crisis for both traders and customers; and insufficient space for future extension of the market for development purposes. During COVID-19 transportation restriction impacted the fish markets system through hampering the supply of farming inputs, trading the mature marketable culture fish and harvested wild fishes in Bangladesh (Sunny et al., 2021). In addition, marketing channel was hit hard by COVID-19 that triggered to disruption of supply chain. Needless to say, not only the activities directly associated with fish marketing but also a great many of other activities which indirectly facilitate fish marketing had been interrupted to a great extent due to the COVID-19 (Fernandez-Gonzalez et al., 2022). For instance, shortage of ice, lesser numbers of fishing equipment, lower man power and thereby insufficient money flow all of these actually results in small amount of fish and fewer purchasers. As, COVID has stigmatized in almost all food production and supply chain that ultimately has impoverished the resources pertain for fish marketing so do the fish availability and all stakeholders. Mandal et al. (2021) suggested economical loss based insurance for the sudden natural shocks like COVID-19. Moreover, rich and large-scale intermediaries related to the fish supply chain can provide financial assistance to the small-scale actors (Mandal et al., 2021; Mamun et al., 2021; Loison et al., 2021). Kamaylo et al. (2021) identified the fish marketing system in Ethiopia having major bottlenecks due to a lack of strong governance and giving adequate information, a lack of credit services and inadequate market integration which are consistent with the value chain of the present study. In addition, Rabbani et al. (2017) noted some problems and constraints related to marine fish marketing in Bangladesh including post-harvest loss, poor physical facilities and transport system, inadequate facilities of wholesale and retail market, lack of ice factories, specialized cold storage, credit facilities, refrigerated vans, qualified staff and market information. Rabbani et al. (2017) and Kaygisiz and Eken (2018) suggested that formal or informal association and providing licensing from Government to fish traders can be effective ways to avoid the common problems in fish markets. As the total available saltwater species in the studied fish markets was lower than that of freshwater species in spite of being demandable. The regular stock of marine and off seasonal freshwater species can be enhanced through smother transportation as well as advanced cold storage for larger stock. However, alternate large market place, reconstruction of market, modern landing center, ice factories, tackling infectious disease and training for all personnel related with fish market are devised considering long term amelioration.

## 5 Conclusion

In the markets of Rajbari Sadar, capture fishes were more common than culture fisheries for being blessed with natural waters including rivers, beel, channels in the Rajbari and for the lower aquaculture practice. In case of demanded marine species and processed products, the market margin of retailers are comparatively higher than that of freshwater fishes due to more intermediaries in marketing channel, transportation and storage. But some existing constraints in the markets influence fish availability, fish quality, fish price, product acceptability, profitability and role of intermediaries in the market. To do so well thought planning from the inception of establishing fish market is prerequisite. Devising a proper design followed by the executions in accordance with that design should be prioritized with utmost concern. Besides, sanitation and COVID-19 are two major issues that could be redressed by enhancing the awareness of pertinent personnel together with providing necessary equipment. There is no denying fact that the marketing channel is a salient reason that incurs cost. In this regard an important approach might be cooperative system which is one of the most functioning bodies in local market of Bangladesh to regulate marketing channels and cost associated with it as well as other mechanisms. Therefore, to prevent low prices during auctions and minimize the marketing expenditures and other problems, the cooperative would help fish producers to sell their products, provide producers requirements, modern economically packaging system and profound transportation passage. Involvement of middlemen in the channel unnecessarily can be controlled by area based licensing or registration of fish traders. Besides, the traders should be well trained on minimizing post-harvest loss of fish and shellfishes which is directly related to consumer acceptability and price value. For ensuring sustainability of fish market well-structured market, smother transportation system, fixed number of fish traders, stability in price, and advanced storage facility will be effective with regular monitoring and guidance of government in combination with other non-governmental organizations.

## Declarations

### Author contribution statement

Zubyda Mushtari Nadia: Conceived and designed the experiments; Performed the experiments; Analyzed and interpreted the data; Wrote the paper.

Prosun Roy, Jakir Hossain, Md. Foysul Hossain, Mofasser Rahman, Md. Abdus Salam & Roksana Jahan: Analyzed and interpreted the data; Contributed reagents, materials, analysis tools or data; Wrote the paper.

### Funding statement

This research did not receive any specific grant from funding agencies in the public, commercial, or not-for-profit sectors.

### Data availability statement

Data will be made available on request.

### Declaration of interest’s statement

The authors declare no conflict of interest.

### Additional information

No additional information is available for this paper.

## Figures and Tables

**ethical Committee:**